# Prevalence and risk factors of brucellosis among febrile patients attending a community hospital in south western Uganda

**DOI:** 10.1038/s41598-018-33915-9

**Published:** 2018-10-18

**Authors:** Richard Migisha, Dan Nyehangane, Yap Boum, Anne-Laure Page, Amaia Zúñiga-Ripa, Raquel Conde-Álvarez, Fred Bagenda, Maryline Bonnet

**Affiliations:** 1 Mbarara University of Science and Technology (MUST), Mbarara, Uganda; 2 Epicentre, Paris, France; 3 Institute for Tropical Health, University of Navarra (ISTUN), Pamplona, Spain; 4 IRD UMI233/, INSERM U1175 Montpellier, France

## Abstract

Human brucellosis, a chronic disease contracted through contact with animals and consuption of unpasteurized dairy products is underreported in limited-resource countries. This cross-sectional study aimed to determine the prevalence and risk factors of brucellosis among febrile patients attending a community hospital in South western Uganda. A questionnaire that captured socio-demographic, occupational and clinical data was administered. Blood samples were tested for *Brucella* antibodies using Rose Bengal Plate Test (RBPT) and blood culture with standard aerobic BACTEC bottle was done. Of 235 patients enrolled, prevalence of brucellosis (RBPT or culture confirmed) was 14.9% (95% CI 10.6–20.1) with a culture confrmation in 4.3% of the participants. The factors independently associated with brucellosis were consumption of raw milk (aOR 406.15, 95% CI 47.67–3461.69); history of brucellosis in the family (aOR 9.19, 95% CI 1.98–42.54); and selling hides and skins (aOR 162.56, 95% CI 2.86–9256.31). Hepatomegaly (p < 0.001), splenomegaly (p = 0.018) and low body mass index (p = 0.032) were more common in patients with brucellosis compared to others. Our findings reveal a high prevalence of brucellosis among febrile patients and highlight a need for implementing appropiate tests, public awareness activities and vaccination of animals to control and eliminate the disease.

## Introduction

Brucellosis is a widespread zoonosis caused by *Brucella* species that can induce considerable human suffering and huge economic losses in livestock^1–3^. The disease is transmitted to humans by contact with fluids from infected animals by several routes such as direct inoculation (cuts and skin abrasions from handling animal carcasses and placent), inhalation of infectious aerosols and ingestion of contaminated milk and meat products^4–6^. The control of brucellosis involves, among other things, implementation of epidemiological surveillance for early detection of cases^7^. Lack of sufficient knowledge about the disease among the physicians, low index of suspicion, under-diagnosis or misdiagnosis have been attributed to wide spread of the disease^8^.

Human brucellosis typically, begins as an acute febrile illness with non-specific flu-like signs that can mimic a variety of acute febrile illnesses^9^. Therefore, laboratory tests are essential for diagnosis but present a particular challenge in settings with limited laboratory capacity and where better-known causes of fever, such as malaria or typhoid, co-occur^10–14^. Thus, it is generally considered that brucellosis is under-diagnosed in many of the areas in which it is endemic^1^. In resource limited and malaria endemic settings, such as Uganda, after exclusion of malaria by rapid tests, fever is often managed based solely on clinical symptoms^15^ using non specific antibiotic regimens that often do not cover *Brucella* species. If left untreated, the disease can progress to osteoarticular, cutaneous, genitourinary, nervous and other complications^16,17^.

Although data are scarce, the existing evidence strongly suggests that brucellosis might be a widespread problem in Africa^12,18,19^. The annual human incidence rate in Uganda was estimated to be 5.8 per 10,000 people^20^. The objective of this study was to determine prevalence and risk factors of brucellosis among febrile patients attending Rushere community Hospital, a cattle keeping area of western Uganda and to compare clinical characteristics between brucellosis and non-brucellosis patients.

## Materials and Methods

### Study setting and population

The cross-sectional study was carried out at Rushere community hospital, Kiruhura district, Western Uganda. Kiruhura District is a farming district where livestock forms the backbone of economic activity. Cow milk and meat are important products produced in the district. The western region is the leading producer of milk in the country per farm, accounting for 33.7% of the total national daily milk production that is estimated at 6.8 million litres^21–23^. Kiruhura district produces more than 100,000 liters of milk daily^22^.

The study participants were patients aged 5 years and above with clinical suspicion of brucellosis defined by a reported or recorded history of fever for a minimum of 7 days and at least one or more of the following criteria: night sweats, headache, weight loss, fatigue, myalgia or arthralgia, anorexia^24^. Participants with other confirmed diagnosis such as smear positive tuberculosis and those who did not consent were excluded from the study. A semi-structured, standardized questionnaire was used to capture socio-demographic characteristics, information on animal exposure and clinical signs.

### Laboratory procedures

The blood tests performed on the sample were Rose Bengal plate test (RBPT), bacterial blood culture, malaria smear microscopy and HIV serology. Additionally, sputum smear microscopy was done in participants with clinical suspicion of tuberculosis. Tests for malaria, HIV and RBPT were done at Rushere community hospital laboratory while tests for bacterial blood cultures were done at Epicentre research laboratory in Mbarara. A minimum of 10 mL of blood sample was collected from each participant using a vacutainer needle: 5 to 7 ml were first collected into blood culture bottle followed by 4 ml in a plain tube for HIV serology and Rose bengal plate test (RBPT). For malaria microscopy, we collected capillary blood from the side of the finger for each participant.

Blood cultures were processed with BACTEC 9240 (Becton Dickinson, Franklin Lakes, USA) in a Biosafety Level 3 (BSL3) laboratory that is maintained at negative pressure. All sample manipulations were performed in a regularly calibrated and certified biosafety cabinet. Between 5 to 7 ml of blood from patients were inoculated in one standard aerobic BACTEC bottle and incubated for seven days and subcultured on *Brucella* base blood agar whenever positive signal occurred by instrument. Suspected colonies were identified by colonial morphology, Gram-staining, standard biochemical procedures (oxidase, catalase, production of H_2_S and urease) and agglutination test using specific antisera. At the end of the first week, bottles not detected positive by the instrument were kept for additional three weeks and subcultures were performed weekly. Cultures were considered negative for *Brucella* in the face of no growth after four weeks of incubation.

Microscopy for malaria parasites was done using Field’s staining technique^25^ for thick blood smears for all participants. HIV testing was done using two rapid tests (Alere Determine™ HIV-1/2, Abbott, Illinois, USA and HIV 1/2 STAT-PAK^®^ Assay,Chembio Diagnostic system, New York, USA) and a third one (Uni-Gold HIV, Trinity Biotech, Co Wicklow, Ireland) in case the first two tests were discordant following the standard national algorithm^26^.

For RBPT, we used two protocols: the standard one and the modified RBPT for testing serum dilutions^27,28^. For the former, 30 µL of plain serum was dispensed on a white glossy ceramic tile and mixed with an equal volume of RBPT antigen (Veterinary Laboratory Agency; England, United Kingdom) using a toothpick. The antigen was previously equilibrated at room temperature and shaken to re-suspend any bacterial sediment. The tile was then rocked at room temperature for 8 minutes (instead of the 4 minutes recommended for animal brucellosis)^29^ and any visible agglutination and/or the appearance of a typical rim was taken as a positive result. For the modified RBPT, positive sera were tested further as follows. Eight 30 µL drops of saline were dispensed on the tile and the first one mixed with an equal volume of the positive plain serum (1/2 serum dilution). Then, 30 µL of this first dilution was transferred to the second drop with the help of a micropipette and mixed to obtain the 1/4 dilution. From this, the 1/8 to 1/128 dilutions were obtained by successive transfers and mixings taking care of rinsing the pipette tip between transfers. Finally, each drop was tested with an equal volume (30 µL) of the RBPT reagent, so that the final dilutions range from 1/2 to 1/256.

Patients diagnosed with brucellosis based on RBPT or blood culture were treated with intravenous gentamycin for two weeks and oral doxycycline for six weeks free of charge. Children below 8 years received cotrimoxazole instead of doxycycline as per Uganda clinical guidelines^30^.

### Sample size and statistical analysis

The sample size of 236 participants was calculated using an estimated prevalence of brucellosis of 15%^31^ with a 5% precision at a 95% confidence interval after a 20% inflation for non-response rate using Epi Info(version 7.1.4.0, CDC, Atlanta US).

Data was entered in EpiData3.1software (EpiData, Odense, Denmark), then exported to STATA version 13 (StataCorp, College Station, Texas, USA) for analysis.

Descriptive analysis of independent variables namely (participant’s age, gender, education level, marital status, religion, income status, occupation, family history of brucellosis, consumption of raw milk and other products) was done. Prevalence of brucellosis was the ratio of the number of patients with fitting definition of probable or confirmed brucellosis by the total number of included patients tested for brucellosis. *A* probable case was a clinically suspected case with a positive RBPT not confirmed by culture and a confirmed case was as a clinically suspected case confirmed by culture and identification of *Brucella* spp.

Chi-square was used to compare categorical variables while continuous variables were compared using student t-test. Wilcoxon rank sum was used to compare nonparametric continuous data. Bivariate analysis was done to evaluate associations between patients’ characteristics and diagnosis of brucellosis (confirmed or probable brucellosis). Covariates associated with *p* value ≤ 0.2) in the bivariate analysis were entered into multivariate logistic regression model through backward stepwise elimination method to obtain the final predictive model of covariates that were independently associated (p < 0.05) with brucellosis.

Ethical approval to conduct the study was obtained from Mbarara University of Science and Technology Research and Ethics Committee (MUST-REC) and the Faculty Research Committee (FRC). The study was given study number 02/03-17 by the REC. We respected the guidelines of Helsinki and CIOMS-2002 (Council for International Organizations of Medical Sciences) regarding research with humans, avoiding any type of physical or moral damage. Written informed consent was obtained by research assistants from all adult participants (18 years and above) and in case of minors (below 18 years) from their parents or guradians. Assent was obtained from children who were older than 7 years.

The datasets generated and analysed during the study are available from the corresponding author on request.

## Results

**Figure 1:**
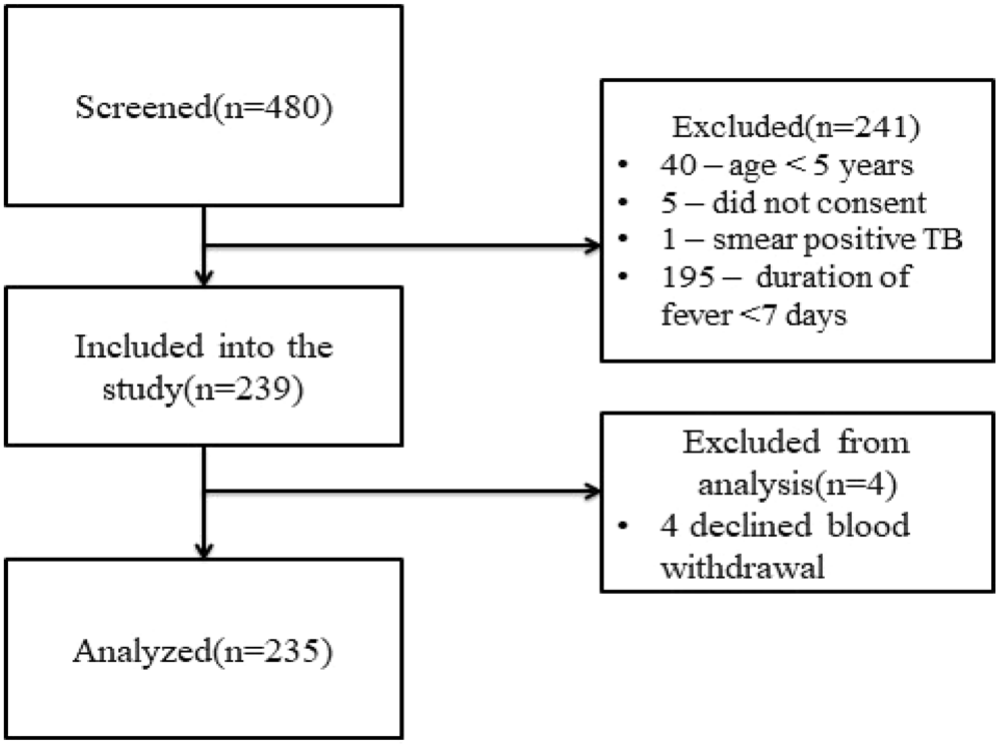
Study profile.

Over a two consecutive months period, out of 480 patients attending the hospital with fever, 239 were recruited and four were secondarily excluded because they subsequently refused blood drawing (Fig. 1). We thus present results from 235 participants enrolled into the study between May and August 2017.

### Patients’ baseline and clinical characteristics

Characteristics of those with and without probable brucellosis are presented in Table 1. The median age of the study participants was 30 years (IQR 22, 45). Participants with probable brucellosis were significantly younger than those without (p = 0.003). Majority of the participants were cattle keepers (56.2%), had acquired formal education (71.5%) and were male (54.0%). The proportion of males with probable brucellosis was 11.5%. The median duration of fever was 9 days (IQR 8,14 days). Fourty-six (19.6%) participants reported history of consumption of raw milk, 3 (1.3%) had history of consumption of raw blood from animals, 54 (23.0%) reported positive history of brucellosis in the family.

**Table 1:** Patients’ demographic and epidemiologic characteristics by diagnosis of brucellosis.

Characteristic, n(%)	Overall N = 235	Probable/confirmed brucellosis N = 35	No brucellosis N = 200	P value
Age in years, median(IQR)	30 (22, 45)	23 (15, 40)	30 (23, 47)	0.012
Age category, years				0.003
<20	40 (17.0)	14 (40.0)	26 (13.0)	
20–29	70 (29.8)	7 (20.0)	63 (31.5)	
30–39	49 (20.9)	5 (14.3)	44 (22.0)	
40–49	30 (12.8)	5 (14.3)	25 (12.5)	
≥50	46 (19.6)	4 (11.4)	42 (21.0)	
Male, sex	127 (54.0)	27 (77.1)	100 (50.0)	0.003
Occupation				
Peasant	67 (28.5)	3 (8.6)	64 (32.0)	0.005
Butcher	4 (1.7)	3 (8.6)	1 (0.5)	0.011
Businessman(woman)	22 (9.4)	0 (0)	22 (11.0)	0.109
Cattle keeper	132 (56.2)	24 (68.6)	108 (54.0)	0.113
Student/pupil	53 (22.6)	18 (51.4)	35 (17.5)	<0.001
Education category				0.271
None	67 (28.5)	6 (17.1)	61 (30.5)	
primary	74 (31.5)	13 (37.1)	61 (30.5)	
secondary	71 (30.2)	14 (40.0)	57 (28.5)	
tertiary	23 (9.8)	2 (5.7)	21 (10.5)	
Type of animal owned				
Cow	181 (77.0)	30 (85.7)	151 (75.5)	0.185
Goat	162 (68.9)	29 (82.9)	133 (66.5)	0.054
Sheep	81 (34.5)	10 (28.6)	71 (35.5)	0.426
Dog	77 (32.8)	16 (45.7)	61 (30.5)	0.077
Pig	16 (6.8)	1 (2.9)	15 (7.5)	0.314
Drinking raw cow’s milk	46 (19.6)	31 (88.6)	15 (7.5)	<0.001
Drinking raw blood	3 (1.3)	2 (5.7)	1 (0.5)	0.059
Handling animals during birth	75 (31.9)	16 (45.7)	59 (29.5)	0.058
History of milking	90 (38.3)	22 (62.9)	68 (34.0)	0.001
Family history of brucellosis	54 (23.0)	20 (57.1)	34 (17.0)	<0.001
Selling hides and skins	3 (1.3)	2 (5.7)	1 (0.5)	0.059
HIV positive	19 (8.1)	2 (5.7)	17 (8.5)	0.577
Malaria positive	37 (15.7)	3 (8.6)	34 (17.0)	0.207
Duration of fever in days, median (IQR)	9 (8, 14)	10 (8, 14)	9 (8, 10.5)	0.041
Antibiotics in past 2 weeks	149 (63.4)	27 (77.1)	122 (61.0)	0.067

IQR: interquartile range.

The most common symptoms overall were headache, joint or back pains and chills. Hepatomegaly (p < 0.001), splenomegaly (p = 0.018) and low body mass index (<18.5 Kg/m^2^) (p = 0.032) were significantly more common among patients with probable brucellosis compared to the others. Other clinical signs and symptoms were similar among patients with and without probable brucellosis (Table 2).

**Table 2:** Clinical characteristics of patients by diagnosis of brucellosis.

Symptom/sign, n (%)	No brucellosis N = 200	Probable/confirmed brucellosis N = 35	p value
Fever (T° ≥ 37.5◦C)	62 (31.0)	16 (45.7)	0.088
Night sweats	10 5 (52.5)	22 (62.9)	0.257
Chills	181 (90.5)	3 2 (91.4)	0.976
Joint or back pain	195 (97.5)	34 (97.1)	0.902
Weight loss	46 (23.0)	9 (25.7)	0.726
Abdominal pain	110 (55.0)	20 (57.1)	0.814
Body weakness	177 (88.5)	34 (97.1)	0.119
Chest pain	87 (43.5)	16 (45.7)	0.563
Cough	48 (24.0)	10 (28.6)	0.407
Muscle pains	164 (82.0)	33 (94.3)	0.069
Headache	186 (93.0)	34 (97.1)	0.355
Splenomegaly	30 (15.0)	11 (31.4)	0.018
Hepatomegaly	5 (2.5)	6 (17.1)	< 0.001
Loss of appetite	120 (60.0)	21 (60.0)	1.000
Constipation	40 (20.0)	11 (31.4)	0.130
Other symptoms	9 (4.5)	3 (8.6)	0.313
Body mass index category (kg/m^2^)			0.032
Less than 18.5	20 (10.0)	9 (25.7)	
18.5–25	105 (52.5)	16 (45.7)	
Above 25	75 (37.5)	1 0 (28.6)	

T°: temperature.

### Prevalence of brucellosis and other co-infections

Thirty five out of the 235 participants had a positive RBPT result and 10 had a blood culture positive for *Brucella* spp giving a prevalence of probable and confirmed brucellosis of 14.9% (95% CI 10.6–20.1) and 4.3% (2.1–7.7), respectively. All confirmed cases were also positive with RBPT and 10/35 (28.6%) of cases with positive RBPT were culture positive (Table 3). None of the cases (21/35) with a RBPT titre of <1:8 had positive results on culture.

**Table 3:** Results of RBPT and blood culture.

RBPT titre, n(%)	Culture negative N = 225	Culture positive N = 10
1:2	9 (4.0)	0 (0)
1:4	12 (5.3)	0 (0)
1:8	2 (0.9)	1 (10.0)
1:16	2 (0.9)	0 (0)
1:32	0 (0)	2 (20)
1:64	1 (0.4)	3 (30.0)
1:128	0 (0)	2 (20)
1:516	0 (0)	2 (20)
Negative	199 (88.4)	0 (0)

RBPT: Rose Bengal Plate Test.

Malaria was diagnosed in 37/235 (15.7%) of the participants. Three out of 35 (8.6%) patients with probable brucellosis also had malaria. HIV prevalence among the participants was 19/235 (8.1%) and 5.7% (2/35) among brucellosis probable or confirmed cases. *Salmonella* Typhi was isolated in one participant. *Cryptococcus neoformans* was also isolated in one participant who was HIV positive.

### Risk factors for probable brucellosis

Consumption of raw milk (adjusted OR – aOR 406.15; 95% CI 47.67–3461.69), history of family member with brucellosis (aOR 9.19; 95% CI 1.98–42.54) and selling of skins and hides (aOR 162.56; 95% CI 2.86–9256.31) were identified as independent risk factors for probable brucellosis. There was no association with the HIV status (Table 4).

**Table 4:** Risk factors for Brucellosis among febrile patients.

Factors n(%)	Probable/confirmed brucellosis	Bivariate	p	Multivariate	p
	n/N (%)	OR 95% CI		aOR 95% CI	
Age category					
<20 yrs	14/40 (35.0)	5.65 (1.68–19.04)	0.005	32.54 (2.12–498.60)	0.012
20–29	7/70 (10.0)	1.17 (0.32–4.23)	0.815	1.93 (0.23–15.85)	0.542
30–39	5/49 (10.2)	1.19 (0 0.30–4.75)	0.802	1.36 (0 0.19–9.56)	0.758
40–49	5/30 (16.7)	2.10 (0 0.52–8.56)	0.301	19.11 (2.08–175.49)	0.009
50 +	4/46 (8.7)	Ref		Ref	
Sex					
Female	8/108 (7.4)	Ref			
Male	27/127 (21.3)	3.38 (1.46–7.79)	0.004	3.88 (0.85–17.58)	0.079
Peasant					
No	32/168 (19.1)	Ref			
Yes	3/67 (4.5)	0.20 (0.06–0.67)	0.01		
Butcher					
No	32/231 (13.9)	Ref			
Yes	3/4 (75.0)	18.66 (1.88–184.92)	0.012		
Cattle keeper					
No	11/103 (10.7)	Ref			
Yes	24/132 (18.2)	1.86 (0.86–4.00)	0.113		
Student/pupil					
No	17/182 (9.3)	Ref			
Yes	18/53 (34.0)	5.00 (2.34–10.64)	<0.001		
Education					
None	6/67 (9.0)	Ref			
Primary school	13/74 (17.6)	2.17 (0 0.59–4.82)	0.141		
Secondary	14/71 (19.7)	2.50 (0.90–6.94)	0.079		
Tertiary	2/23 (8.7)	0.97 (0.18–5.17)	0.97		
Owning a cow					
No	5/54 (9.3)	Ref			
Yes	30/181 (16.6)	1.95 (0 0.72–5.29)	0.192		
Owning a goat					
No	6/73 (8.2)	Ref			
Yes	29/162 (17.9)	2.43 (0.96–6.15)	0.06		
Owning a dog					
No	19/158 (12.0)	Ref			
Yes	16/77 (20.8)	1.92 (0 0.92–3.98)	0.08		
Owning sheep					
No	25/154 (16.2)	Ref			
Yes	10/81 (12.4)	0.73 (0.33–1.60)	0.428		
Owning a pig					
No	34/219 (15.5)	Ref			
Yes	1/16 (6.3)	0.36 (0 0.05–2.84)	0.334		
Drinking raw milk					
No	4/189 (2.1)	Ref			
Yes	31/46 (67.4)	95.58 (29.76–306.95)	<0.001	406.15 (47.67–3461.69)	<0.001
Drinking raw blood					
No	33/232 (14.2)	Ref			
Yes	2/3 (66.7)	12.06 (1.06–136.80)	0.044		
Handling animals during birth					
No	19/160 (11.9)	Ref			
Yes	16/75 (21.3)	2.01 (0 0.97–4.18)	0.061		
History of milking					
No	13/145 (9.0)	Ref			
Yes	22/90 (24.4)	3.29 (1.56–6.92)	0.002		
Family history of brucellosis					
No	13/155 (8.4)	Ref			
Yes	20/54 (37.0)	6.43 (2.91–14.19)	<0.001	9.19 (1.98–42.54)	0.005
Do not know	2/26 (7.7)				
Selling hides and skins					
No	33/234 (14.2)	Ref			
Yes	2/3 (66.7)	12.06 (1.06–136.80)	0.044	162.56 (2.86–9256.31)	0.014
HIV status					
Negative	33/216 (15.3)	Ref			
Positive	2/19 (10.5)	0.65 (0 0.14–2.96)	0.58		

OR: odds ratio; aOR: adjusted odds ratio; CI: Confidence interval; Ref: Reference category.

## Discussion

Our results show a high prevalence of probable brucellosis among patients consulting for fever in this hospital based survey (14.9%). This is close to the prevalence of 13.3% among febrile patients in Kampala^31^ and is consistent with the high prevalence reported in general population in rural area in Uganda (11.7% and 13.4%)^32,33^ or among butchers in Mbarara (7%) and Kampala (12%) districts^34^.

Outside Uganda, a similar hospital based study in a predominantly pastoral community in nearby Kenya indicated a comparable high sero-prevalence (13.7%)^35^ among febrile patients, and 32.5% in Nigeria^36^, highlighting brucellosis as an important cause of fever. The prevalence of probable cases in our study is however higher than that reported among febrile patients in other countries: 7% in Egypt (p < 0.001)^37^; 7.7% in Mali (p = 0.03)^38^. This is probably because of the high prevalence of brucellosis in livestock in Western Uganda (55.6%)^39^. Indeed, a strong association has been reported between prevalence of the disease in animals and in humans^40^. This variation from our findings can also perhaps be attributed to variations in different serodiagnostic approaches to the disease. In this particular study, we used the RBPT whose sensitivity and specificity vary from 87% to >99%^27,28,41,42^.The fact that vaccination of animals against brucellosis in Uganda using *Brucella abortus* S19 vaccine is a voluntary exercise^43^ and is not routinely done, most likely because of economic and logistic reasons^18^,may further explain the high prevalence of the disease.

The prevalence of culture confirmed brucellosis reported in our study of 4.3% is similar to that reported among hospitalized febrile patients in Northern Tanzania of 3.5%^14^. In our study, the culture positivity was 28.6% among the brucellosis probable cases. This is comparable to the one reported in the study in Jordan of 23.4%^44^ but lower than the culture positivity of 74.1% reported in Kuwait^45^. The lower culture positivity in our study might be attributed to high proportion of study participants who received antibiotics prior to hospital visit (63.4%). All patients with positive cultures in our study had positive serology results with titres ≥1:8 on RBPT, contrary to other studies that have reported positive cultures with negative serological test results^46^. Although the study was not powered to look at the association between the titer level of the serology and the culture results, this supports the proposed cut-off of 1:8 by Diaz *et al*.^28,47^.

Our findings also show that consumption of raw cow milk, history of brucellosis in a family member and selling cattle’s hides and skins are independent risk factors of brucellosis. This is in agreement with findings from other studies^7,48–50^. Therefore, in high brucellosis burden countries, individuals selling hides and skins are at risk of occupational exposure^7^. Consumption of raw milk is also associated with higher rates of recurrent disease^7,51^. Consistent with our findings, the existence of another infected family member is a well known major risk factor for brucellosis^7,48^. This is because families are likely to share a common infected food source^7^.

Most clinical signs and symptoms were similar among patients with brucellosis and those without brucellosis except hepatomegaly and splenomegaly that were more common in probable or confirmed brucellosis cases(p < 0.05), in agreement with findings from other studies^35,46,52–54^. This is due to persistent bacterial colonization of reticuloendothelial system^54,55^. As previously reported, we did not find an association between HIV status and the risk of brucellosis^56,57^.

This study had some limitations: first, it was done in one hospital in one district and so the findings may not be generalizable to the entire region; second, the study was based on relatively small numbers which impacted on some results of the multivariate analysis that had very wide confidence intervals; third, the study may also be prone to recall bias since some of the data came from self-reporting and participants were asked for information about exposure to animal products that could have taken place longer period preceding the interview date (more than one month).

## Conclusions

Brucellosis is frequent among patients with prolonged fever attending Rushere Community Hospital mostly due to high exposure within this community from cultural practice regarding consumption of raw milk in the Western region of Uganda. Based on our findings, we recommend that patients with fever for at least one week, living at high risk of exposure should be routinely screened for brucellosis for early diagnosis and prompt treatment. This may require the development of rapid, affordable and easy to use point of care tests in areas where people are more exposed. Our findings also highlight the need for vaccination of animals, carrying out public education activities to sensitize the community on boiling and/or pasteurization of milk before consumption, and use of personal protective gear while handling animal as well as screening of family members of brucellosis cases for early disease detection in order to control and eliminate the disease.
